# Automatic Speech Recognition from Neural Signals: A Focused Review

**DOI:** 10.3389/fnins.2016.00429

**Published:** 2016-09-27

**Authors:** Christian Herff, Tanja Schultz

**Affiliations:** Cognitive Systems Lab, Department for Mathematics and Computer Science, University of BremenBremen, Germany

**Keywords:** ASR, automatic speech recognition, ECoG, fNIRS, EEG, speech, BCI, brain-computer interface

## Abstract

Speech interfaces have become widely accepted and are nowadays integrated in various real-life applications and devices. They have become a part of our daily life. However, speech interfaces presume the ability to produce intelligible speech, which might be impossible due to either loud environments, bothering bystanders or incapabilities to produce speech (i.e., patients suffering from locked-in syndrome). For these reasons it would be highly desirable to not speak but to simply envision oneself to say words or sentences. Interfaces based on imagined speech would enable fast and natural communication without the need for audible speech and would give a voice to otherwise mute people. This focused review analyzes the potential of different brain imaging techniques to recognize speech from neural signals by applying Automatic Speech Recognition technology. We argue that modalities based on metabolic processes, such as functional Near Infrared Spectroscopy and functional Magnetic Resonance Imaging, are less suited for Automatic Speech Recognition from neural signals due to low temporal resolution but are very useful for the investigation of the underlying neural mechanisms involved in speech processes. In contrast, electrophysiologic activity is fast enough to capture speech processes and is therefor better suited for ASR. Our experimental results indicate the potential of these signals for speech recognition from neural data with a focus on invasively measured brain activity (electrocorticography). As a first example of Automatic Speech Recognition techniques used from neural signals, we discuss the *Brain-to-text* system.

## 1. Introduction

With services like Siri and Google Voice Search, speech-driven applications arrived in our daily life and are used by millions of users every day. These speech interfaces allow for natural interaction with electronic devices and enable fast input of texts. **Brain-computer interfaces (BCIs)** (Wolpaw et al., 2002) on the other hand are currently only used by a small number of patients (Vaughan et al., 2006). This is in part due to the unnatural paradigms which have to be employed to enter commands or texts via the BCI. Motor imagery based BCIs (McFarland et al., 2000) use imagined movement of hands, arms or feet to issue directional commands. To spell out texts, users often have to focus on a single letter at a time which is then selected (Farwell and Donchin, 1988; Sutter, 1992; Donchin et al., 2000; Müller-Putz et al., 2005). Even though these are the fasted currently known BCIs, they are still rather slow and very unnatural. Using speech as a paradigm for BCIs would solve these problems and enable very natural communication. A BCI based on speech would enable communication without the need for acoustic voice production, while maintaining the same advantages as ordinary speech interfaces. Brain activity is not the only approach possible for silent speech interfaces, see the review (Denby et al., 2010) for a description of other approaches to silent speech interfaces. However, only silent speech interfaces based on brain activity would enable severely disabled persons (i.e., locked-in syndrome) to communicate with the outside world.

KEY CONCEPT 1 **Brain-computer interfaces (BCIs)**

A Brain-Computer Interface is a system which sends messages or commands to a computer without using the brain's normal output pathways of peripheral nerves and muscles.

The intention of this focused review is to investigate the potential of neural signals—captured by different brain imaging techniques—as input for **Automatic Speech Recognition (ASR)**. Brain imaging techniques can be broadly divided into two categories. Imaging methods based on metabolic processes measure the amount of oxygenated and/or deoxygenated blood in certain areas of the brain. We will discuss functional Magnetic Resonance Imaging (fMRI) and functional Near Infrared Spectroscopy (fNIRS) from this category of imaging techniques, as they are the most commonly used in neuroimaging.

KEY CONCEPT 2 **Automatic Speech Recognition (ASR)**

Automatic Speech Recognition is a technology that enables the recognition of spoken language into a textual representation by computers. These technologies often rely on statistical models like Hidden-Markov-Models and can now be found in a large variety of consumer electronics from cars to mobile phones.

Measurement of electric potentials is possible both on the scalp and invasively. We will be discussing electroencephalography (EEG) and magnetoencephalography (MEG) as non-invasive and electrocorticography (ECoG) and microarrays as invasively measured examples of electrophysiological signals.

### 1.1. Metabolic signals

Brain imaging techniques based on metabolic processes measure the amount of oxygen-carrying blood in certain areas of the brain. Active neurons have a higher demand for energy in the form of oxygen, resulting in increased blood flow to these active regions to satisfy the increased demand. Thus, the amount of fresh oxygenated blood can be used as an indirect marker of neural activity in very small regions, called voxels. Blood vessels form a very intricate network in the brain and can thus regulate the supply to very specific regions in the brain. Brain imaging techniques based on metabolic processes can therefor measure activity with a very high spatial resolution. On the flip side, these metabolic processes are slow in nature and take several seconds to complete. Continuous speech processes, like the production of single vowels or consonants, happen as fast as 50 ms, which makes them impossible to be measured with metabolic-based imaging techniques.

#### 1.1.1. Functional magnetic resonance imaging

Hemoglobin, the oxygen carrying part of the blood, has different magnetic properties when oxygenated or deoxygenated. These different properties can be detected by the strong magnetic fields produced in the large tube of the MRI. Observing the changes in these relative hemoglobin concentrations allows for the estimation of neural activity in a voxel. fMRI is instrumental in a large variety of neuroimaging studies. The high spatial resolution over the entire brain enables detailed investigations of neural processes during all sorts of cognitive processes.

The inherently slow natures of metabolic processes rule out fMRI to be used for continuous speech recognition, as phones change much too quick for the slow hemodynamic responses. However, fMRI can be used in neuroscientific studies to learn more about speech perception, speech production and reading. See the excellent reviews (Price, 2012; Talavage et al., 2014) for more on this topic. Besides neuroscientific breakthroughs, it has been shown that fMRI recordings can be used to classify isolated **phones** or attended speaker (Formisano et al., 2008).

KEY CONCEPT 3 **Phone**

A phone is a distinct speech sound that can be perceptually differentiated from other speech sounds.

Moreover, the sheer size and cost of the apparatus and the fact that subjects have to remain motionless in it for extended periods of time make it ill-suited for real-life interfaces. Nevertheless, fMRI studies are indispensable for neuroscience, due to their unparalleled spatial resolution.

#### 1.1.2. fNIRS

Light in the near infrared part of the light spectrum (~700–900 nm) disperses through skin, bones and tissue, but is absorbed by hemoglobin. It can be used to indirectly estimate brain activity by shining it through the skull and measuring how much of the re-emerging light is attenuated. The more light is absorbed, the more oxygenated hemoglobin and thus the more active the specific brain region. fNIRS measures similar physiological signals as fMRI with much cheaper devices, which can be head-mounted and do not require the subject to lay motionless. It provides signals on the same temporal scale as fMRI measurements, but with a far coarser spatial resolution. Additionally, fNIRS is only able to measure the hemodynamic response in outer areas of the cortex and is not able to provide signals from the entire brain.

While fNIRS can be used for BCIs both for direct control (Coyle et al., 2007; Sitaram et al., 2007) and passive monitoring of user states (Heger et al., 2013; Herff et al., 2013b, 2015a; Heger et al., 2014; Hennrich et al., 2015), it is not well suited for ASR, as recorded processes are far too slow to capture the fast dynamics of speech.

To investigate speech processes with fNIRS, some studies (Herff et al., 2012a,b, 2013a) discriminated the type of speech production that a user currently undertook, such as audible speech, silently-mouthed speech and speech imagery. These studies show that fNIRS can be used to study speech processes in the brain, but is not suitable for continuous speech recognition from neural signals.

### 1.2. Electrophysiological signals

Measurement of electrophysiological signals from the brain can be carried out both invasively or non-invasively. Electrodes can either measure ensembles of neurons firing in synchrony, which is done by MEG, EEG, and ECoG, or needle electrodes can be used to measure single action potentials (spikes) from individual neurons. Obviously the spatial and temporal resolution of single neuron measurements using microarrays is unparalleled, but it comes at the disadvantage of only covering small areas and thus not measuring all areas involved in speech production. MEG, EEG, and ECoG can cover larger areas or even the entire brain, but with coarser spatial resolution.

#### 1.2.1. Microarrays

Microarrays provide high resolution information of very small brain areas with a size of few square milimeters. The spatial and temporal resolution down to single action potentials is unparalleled. Microarrays in the speech-motor cortex have successfully been used to decode intended phone production (Brumberg et al., 2011) for a number of isolated phones or to synthesize vowels (Guenther et al., 2009; Brumberg et al., 2010). As microarrays cover only very small areas of the cortex, they might miss crucial information from other parts of the brain involved in the speech production process and might thus not be well suited in the combination with ASR technology.

#### 1.2.2. Electroencephalography (EEG)

Electroencephalography measures electric potentials of large ensembles of neurons firing at the same time by placing electrodes on the scalp. With these scalp electrodes, experiments are easy to setup and do not require a clinical environment. EEG is the de-facto standard for BCIs as the technique is non-invasive and easy to setup, while still providing high-quality signals with good temporal resolution.

However, the placement on the scalp makes EEG very prune to motion artifacts, especially from head movements. Muscle movements in the face as appearing from spoken speech yield large electromyographic and glossokinetic artifacts in the EEG that are not produced by brain activity. In fact, EMG activity in facial muscles alone can be used to accurately decode speech by itself (Schultz and Wand, 2010; Herff et al., 2011). Additionally, due to volume conduction effects, each EEG electrode measures signals from a variety of superimposed sources, making localization of brain activity very difficult.

While EEG is the de-facto standard for current BCIs, it can currently not be used for ASR from neural signals, as the first step for speech interfaces, namely speech decoding from audible speech is not possible due to artifact contamination. However, studies have used EEG successfully to investigate perceived speech (Di Liberto et al., 2015; O'sullivan et al., 2015) or to classify limited numbers of imagined isolated phones (Yoshimura et al., 2016).

#### 1.2.3. Magnetencephalography (MEG)

Magnetencephalography measures synchronized activity of large groups of neurons using magnetometers placed around the head, requiring extensive magnetic shielding around the device. MEG provides high temporal and acceptable spatial resolution and is less distorted by the scalp than EEG. However, movement, especially of the facial muscles yield large artifacts in the MEG signals, it is thus difficult to investigate overt speech production with MEG.

The high spatial and temporal resolution of MEG allow for thorough investigation of speech process, including the comparison between speech production and perception (Houde et al., 2002) and the comparison of processing of phonetic and musical sounds (Tervaniemi et al., 1999). Heinks-Maldonado et al. (2006) presented evidence for a forward model in speech production. MEG has been used for classification of speech processes, Guimaraes et al. (2007) showed single trial classification between two aurally presented words, but is difficult to be used with overt speech production, as would be needed for ASR.

Due to the large chambers needed for MEG devices, they are not ideally suited for future prosthetic devices.

#### 1.2.4. Electrocorticography (ECoG)

Electrocorticography measures electrical potentials directly on the brain surface. ECoG grids are normally used in the process of epilepsy surgery and are not originally intended for neuroscientific studies or BCIs. ECoG provides high spatial and high temporal resolution while not being affected by motion or glossokinetic artifacts. It provides signals unfiltered by scalp and skin. Electrode positions are usually within 1 cm or less from each other and thus provide high-density neural recordings from large areas of the cortex. These characteristics make ECoG ideally suited for the investigation of speech, as artifacts of natural speech production do not affect the neural recordings. ECoG has been used to investigate the differences between speech production and perception (Cheung et al., 2016). Neural representations of phonetic features during speech production are documented in Chang et al. (2010) and Mesgarani et al. (2014).

Isolated aspects of speech have successfully been decoded. Lotte et al. (2015) demonstrated that phonetic features can be decoded from ECoG data. Syllables (Bouchard and Chang, 2014) and isolated words (Kellis et al., 2010) were shown to be distinguishable from neural data. Extending upon these ideas, Mugler et al. (2014) showed that a complete set of manually labeled phones can be classified from ECoG recordings.

An alternative approach to ASR from neural signals is the reconstruction of the acoustic waveform from neural signals. This would allow users to produce normal acoustic speech from imagined speech, which would be the most natural way to restore communication for locked-in patients. For other applications, such as human-computer interaction, recognition of a textual representation is better suited as a waveform would disturb bystanders and would have to be recognized by the computer. Pasley et al. (2012) have shown that perceived speech could be reconstructed from ECoG recordings. Martin et al. (2014) showed that the spectrogram of spoken speech can be reconstructed from ECoG. See Chakrabarti et al. (2015) for a review on speech decoding and synthesis from ECoG.

The combination of the ideal characteristics of ECoG for ASR—such as high temporal and spatial resolution, robustness toward artifacts and being unfiltered by skull and scalp—together with the rich literature on speech processes investigated using ECoG make ECoG and ideal candidate to be used for ASR from neural signals. In our *Brain-to-text* study (Heger et al., 2015; Herff et al., 2015b) we could show that ECoG could indeed be used to decode continuously spoken speech from neural signals.

## 2. Materials and methods

In our *Brain-to-text* study (Herff et al., 2015b), we obtained data from seven patients undergoing surgery for epilepsy treatment. The treatment required the patients to have electrode grids implanted on the brain surface. Each patient had very different placement of the grids depending on his or her clinical needs. The electrode grids stay implanted for periods between a few days and a couple of weeks and patients agreed to take part in our experiment during this time.

**Figure 1:**
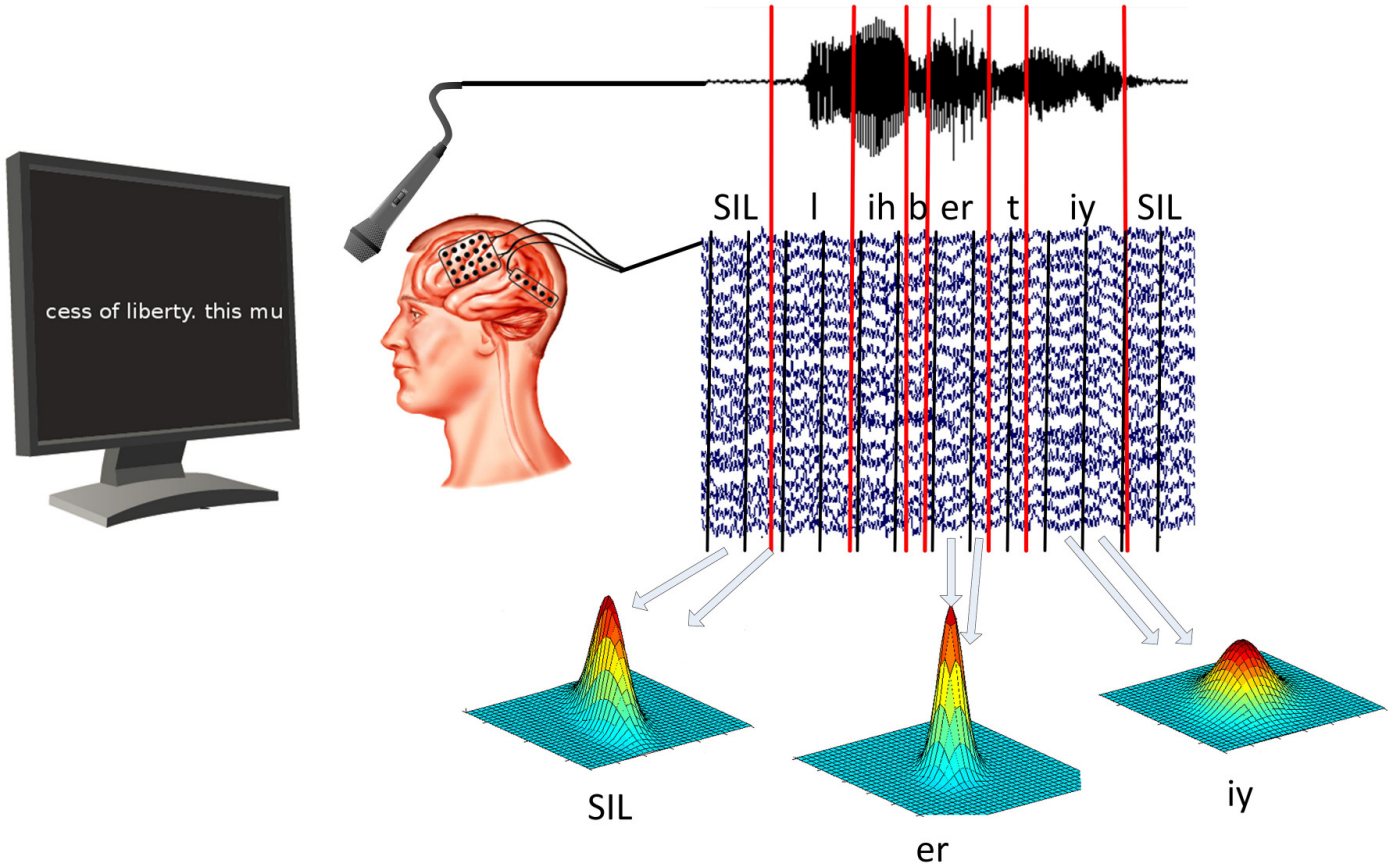
**ECoG and audio data are recorded at the same time**. Speech decoding software is then used to determine timing of vowels and consonants in acoustic data. ECoG models are then trained for each phone individually by calculating the mean and covariance of all segments associated with that particular phone.

In our experiment, patients were asked to read out texts that were shown on a computer screen in front of them. Texts included political speeches, fan-fiction and children rhymes. While the participants read the text, ECoG data and acoustic data were recorded simultaneously using BCI2000 (Schalk et al., 2004). All patients gave informed consent to participate in the study, which was approved by the Institutional Review Board of Albany Medical College and the Human Research Protections Office of the US Army Medical Research and Materiel Command. Once the data was recorded, we used ASR software (Telaar et al., 2014) to mark the beginning and ending of every spoken phone. See Figure 1 for a visualization of the experiment setup.

To extract meaningful information from the ECoG data, we calculated logarithmic broadband gamma power between 70 and 170 Hz. Gamma power has been shown to contain highly localized task specific information (Miller et al., 2007; Leuthardt et al., 2011; Pei et al., 2011; Potes et al., 2012). As ECoG data and acoustic data are recorded simultaneously, we can use the timings of the phones in the neural data, as well. This enables us to calculate an ECoG phone model for the prototypical neural activity related to each individual phone. This prototypical activity is characterized by the mean and covariance of gamma power for each selected electrode and temporal offset. The best temporal offsets and electrodes are selected on the training data using the discriminability between phones as a criterion. Figure 1 illustrates the training process for **ECoG phone models**.

KEY CONCEPT 4 **ECoG Phone Models**

ECoG phone models can be used to estimate the likelihood that an internal of ECoG activity is a certain phone. This generative models might for example return that newly recorded data have a probability of 0.6 of being a /l/, but only a probability of 0.1 of being a /b/.

**Figure 2:**
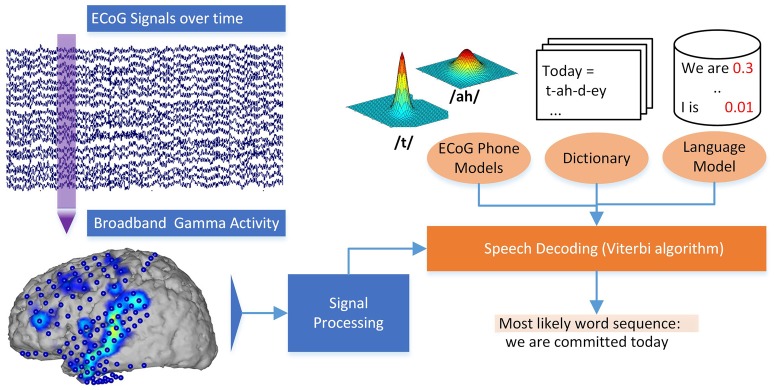
**Decoding process in the ***Brain-to-text*** system**. Broadband gamma power is extracted for a phrase of ECoG data. The most likely word sequence is then decoded by combining the knowledge of ECoG phone models, dictionary and language model.

These models for each phone can be used to estimate the likelihood of a certain phone given a piece of ECoG data. Additionally, the calculated generative models for each phone can be used to gain insights into the neural basis of speech production for different phones. Even though these ECoG phone models alone could be used to pick the most likely phone for each interval of ECoG activity, ASR software works by adding crucial information through a statistical **language model** (Jelinek, 1997; Stolcke, 2002) and a pronunciation **dictionary**. The combination of these three ingredients yields the great results known from speech interfaces. The ASR software extracts the search result by identifying the sequence of words from the dictionary that has the best score combination from language model and the ECoG phone models. Using these ideas from ASR, our *Brain-to-text* system is able to create a textual representation of spoken words from neural data. See Figure 2 for a graphical explanation of the decoding process.

KEY CONCEPT 5 **Language Model**

A language model estimates how likely a word is given the preceding words. In N-gram language modeling, this is done by calculating probabilities of single words and probabilities for predicting words given the history of *n*−1 previous words. The language model would thus contain that “I am” is very likely, while “I is” is rather unlikely.

KEY CONCEPT 6 **Dictionary**

A pronunciation dictionary contains the mapping of phone sequences to words, for example, describing that the word liberty comprises of the phone sequence “/l/ /ih/ /b/ /er/ /t/ /iy/.” The dictionary is used to guide the search for the correct words in ASR, as only words included in the dictionary can be recognized.

## 3. Results

We evaluated our *Brain-to-text* system by training the phone models on all but one spoken phrase of a participant and then decoding the last remaining, unknown phrase. This procedure is repeated so that each phrase is decoded once. As electrode montages and brain physiologies are very different between participants, the ECoG phone models are trained for each participant individually. Acoustic speech recognition systems are trained on thousands of hours of data, while only a few minutes have to suffice for our system. To correct for this very limited amount of data, we evaluate our system with only between 10 and 100 words that can be recognized (i.e., that are in the dictionary).

For 10 words in the dictionary, we achieved up to 75% of correct words, meaning that in a phrase of 10 words, only 3 words were wrong or at the wrong position. When the system could choose between 100 words, still 40% of words were placed correctly at the appropriate position in a sentence. We used randomization tests to check whether this results were better than guessing and could show that all results were better than chance. Breaking down the decoded phrases further, we could show that on average, up to 54% of the ECoG intervals were assigned the correct phone. When looking at true positive rates for each phone, it was shown that each phone yielded better than chance true positive rates. This means that all phones worked reliably and that decoding was not based on the detection of a small subset of phones.

This results show that applying ASR to neural data is possible when the participant is speaking loudly. This is a first step toward ASR from imagined speech processes, but there are still a lot of challenges until imagined continuous speech can be decoded into a textual representation. While speech production and imagined speech production might yield similar neural responses in brain motor areas and speech planning areas, the observed neural activity in the brain's auditory cortex is distinctly different, as participants do not hear their own voice when only imagining to speak.

## 4. Conclusion and discussion

In this focused review, we argue why only few brain imaging techniques can be used for ASR to produce textual representations from imagined words. While no reconstruction of continuously imagined speech to a textual representation has been shown yet, we argue that measurement techniques based on electrophysiological signals are generally better suited than those based on metabolic processes. We show that ECoG is the most promising technique and demonstrate how audibly spoken speech can be recognized from ECoG data using ASR technology in our *Brain-to-text* system. Despite these first promising results, there still are a lot of open research questions to be addressed before neuroprostheses based on imagined speech processes become a reality. While having a lot of similar characteristics, imagined speech production is also distinctly different form overt speech yielding challenges for future decoding approaches. Also, initial alignment for model training is very difficult, when no audible waveform for alignment is present. These challenges need to be solved before ASR can be applied to neural signals for real life applications.

Besides the direct implications for neural prothesis based on speech processes, the successful results of the *Brain-to-text* system show promises for other areas, as well. The *Brain-to-text* systems demonstrates that leveraging advanced technology from non-adjacent areas can drastically increase decoding performance and enable new paradigms. Without the refined decoding approaches and knowledge sources from the Automatic Speech Recognition community, the results in our study could not present the entire decoding pipeline from neural signals to textual representation of words.

For neuroscience, the single trial analysis approach utilized in BCI and *Brain-to-text* yield resilient results without the need to aggregate large cohorts. Especially usage of generative models yields easily interpretable models that can grant important insights into complex brain functions without typical statistical problems associated with large numbers of variables (Eklund et al., 2016).

A fear often associated with BCI in general and the speech decoding in *Brain-to-text* in particular is that private thoughts could be read and thereby freedom of thought not be guaranteed any longer. In *Brain-to-text* activations associated with the production of speech are decoded, from planning to articulate speech prior to voice onset, to control of facial muscles, to processing of heared sounds. Thought processes or internal voice, while being formulated in words as well, do not make use of areas associated with the movement of articulatory muscles. So even if neural prothesis based on imagined speech processes become a reality, there is still a large distinction between thought processes and the process of imagining oneself to speak.

## Author contributions

CH wrote the manuscript. TS supervised the research and revised the manuscript.

### Conflict of interest statement

The authors declare that the research was conducted in the absence of any commercial or financial relationships that could be construed as a potential conflict of interest.
